# Cheminformatics Analysis of the Multitarget Structure–Activity Landscape of Environmental Chemicals Binding to Human Endocrine Receptors

**DOI:** 10.1021/acsomega.3c07920

**Published:** 2023-12-13

**Authors:** Shanmuga Priya Baskaran, Ajaya Kumar Sahoo, Nikhil Chivukula, Kishan Kumar, Areejit Samal

**Affiliations:** † The Institute of Mathematical Sciences (IMSc), Chennai 600113, India; ‡ Homi Bhabha National Institute (HBNI), Mumbai 400094, India

## Abstract

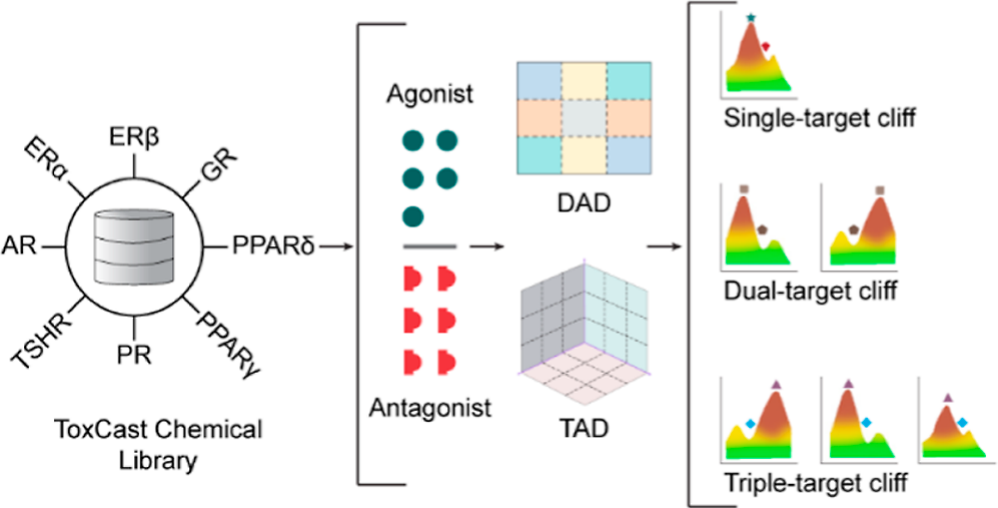

In human exposome, environmental chemicals can target and disrupt different endocrine axes, ultimately leading to several endocrine disorders. Such chemicals, termed endocrine disrupting chemicals, can promiscuously bind to different endocrine receptors and lead to varying biological end points. Thus, understanding the complexity of molecule–receptor binding of environmental chemicals can aid in the development of robust toxicity predictors. Toward this, the ToxCast project has generated the largest resource on the chemical–receptor activity data for environmental chemicals that were screened across various endocrine receptors. However, the heterogeneity in the multitarget structure–activity landscape of such chemicals is not yet explored. In this study, we systematically curated the chemicals targeting eight human endocrine receptors, their activity values, and biological end points from the ToxCast chemical library. We employed dual-activity difference and triple-activity difference maps to identify single-, dual-, and triple-target cliffs across different target combinations. We annotated the identified activity cliffs through the matched molecular pair (MMP)-based approach and observed that a small fraction of activity cliffs form MMPs. Further, we structurally classified the activity cliffs and observed that R-group cliffs form the highest fraction among the cliffs identified in various target combinations. Finally, we leveraged the mechanism of action (MOA) annotations to analyze structure–mechanism relationships and identified strong MOA-cliffs and weak MOA-cliffs, for each of the eight endocrine receptors. Overall, insights from this first study analyzing the structure–activity landscape of environmental chemicals targeting multiple human endocrine receptors will likely contribute toward the development of better toxicity prediction models for characterizing the human chemical exposome.

## Introduction

The human exposome encompasses exposure to all environmental factors, and understanding the adverse effects of such exposures on human health is a key goal of environmental science in the 21st century.^1^ In particular, certain environmental chemicals have been observed to interact and disrupt the normal functioning of the human endocrine system and are termed as endocrine disrupting chemicals (EDCs).^2−4^ EDCs can target several endocrine axes or organs, where their selective binding to different endocrine receptors leads to different adverse outcomes such as metabolic, reproductive, and neurological disorders and cancers.^2−6^ Therefore, analysis of the multimodal nature of EDC–receptor binding can enable us to link the various adverse effects caused by the EDCs.

In pharmacology, the concept of promiscuity in molecule binding has aided in a better understanding of drug targets and in the design of novel polypharmacological drugs.^7,8^ A similar understanding of the complexity in molecule–receptor binding of toxic chemicals^9^ can aid in the development of robust toxicity predictors. In this direction, the ToxCast project^10^ has screened nearly 10,000 environmental chemicals across multiple human receptors and generated several chemical–receptor activity data sets.^11,12^ However, the heterogeneity in the structure–activity landscape of chemicals targeting multiple receptors has not been explored in the ToxCast chemical space.

The methodology to analyze the heterogeneity in the structure–activity landscape of chemicals has been extensively developed for a single-target chemical space,^13−17^ but similar efforts are limited for the multitarget chemical space. In particular, Bajorath and colleagues have primarily used network-like representations to analyze the promiscuous chemicals in the context of drug discovery research.^18−23^ Independently, Medina-Franco and colleagues had extended the concept of the structure–activity similarity (SAS) map to identify multitarget activity cliffs in the drug-relevant chemical space.^24^ They proposed activity difference maps [dual-activity difference (DAD) and triple-activity difference (TAD) maps] to analyze and identify the single-, dual- and triple-target cliffs present in the chemical space.^25,26^ Importantly, these activity difference maps aid in the comparison in the direction of the structure–activity relationship (SAR) among chemicals forming multitarget activity cliffs. However, such a methodology has not been used to analyze the multitarget toxic chemical space.

In this study, we leveraged the activity difference map-based approach to analyze the structure–activity landscape of chemicals targeting several human endocrine receptors. To achieve this, we systematically retrieved the chemical activity values and the corresponding biological end points across eight human endocrine receptors from ToxCast. We employed both the DAD and TAD maps to analyze and identify the single-, dual-, and triple-target cliffs across chemicals targeting all combinations of receptors. Further, we used the matched molecular pair (MMP)-based approach to annotate the identified activity cliffs. Subsequently, we leveraged the structural information to classify the chemical pairs forming activity cliffs. We also analyzed the heterogeneity in the structure–mechanism relationships of chemicals targeting the different receptors and identified mechanism of action (MOA) cliffs. In summary, the present study is the first attempt in analyzing the heterogeneity in the structure–activity landscape of toxic chemicals targeting multiple human endocrine receptors.

## Results and Discussion

### Exploration of the Structure–Activity Landscape of Chemicals Targeting Multiple Human Endocrine Receptors

Our main objective is to analyze the heterogeneity in the structure–activity landscape of chemicals targeting multiple human endocrine receptors. To achieve this, we systematically obtained the agonist and antagonist chemical data from the ToxCast project for eight human endocrine receptors (Table S1), namely, androgen receptor (AR), estrogen receptor alpha (ERα), estrogen receptor beta (ERβ), glucocorticoid receptor (GR), peroxisome proliferator-activated receptor delta (PPARδ), peroxisome proliferator-activated receptor gamma (PPARγ), progesterone receptor (PR), and thyroid stimulating hormone receptor (TSHR) (Methods section and Tables S2 and S3). We further generated 28 dual-target combinations (Tables S4 and S5) and 56 triple-target combinations (Tables S6 and S7) in each of the agonist and antagonist data sets (Methods). We considered structurally similar chemicals in each of these combinations for further analysis (Methods section).

Among the 28 dual-target agonist data sets, we observed that the activity values (pAC_50_) of chemicals targeting both GR and PPARγ showed the highest correlation (Pearson coefficient 0.75), while those targeting TSHR and ERβ showed the lowest correlation (Pearson coefficient −0.16) (Table S4). This suggests that the structure–activity landscapes of chemicals targeting GR and PPARγ are more similar than for other pairs of targets. Similarly, among the antagonist data sets, we observed that the activity values of chemicals targeting PPARδ and PPARγ showed the highest correlation (Pearson coefficient 0.8), while those targeting PPARδ and ERβ showed the lowest correlation (Pearson coefficient −0.04) (Table S5), suggesting that chemicals targeting PPARδ and PPARγ have similar structure–activity landscapes.

### Identification of Single-, Dual- and Triple-Target Activity Cliffs among Chemicals in the Agonist Data Set

**Figure 1:**
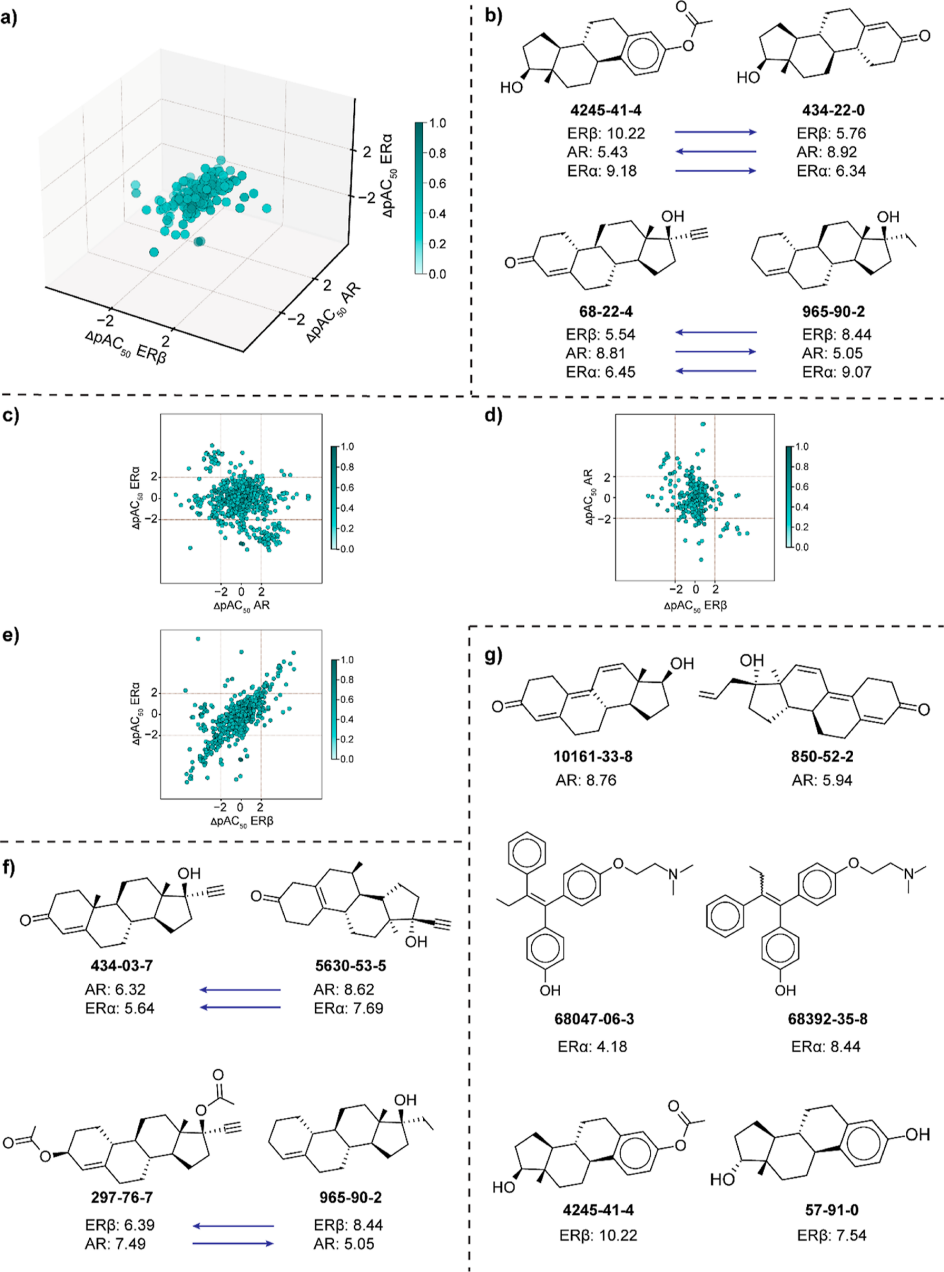
Analysis of the structure–activity landscape of chemicals in the agonist data set. (a) TAD map for common and structurally similar chemicals targeting AR, ERα, and ERβ receptors. Each axis of the TAD map represents the values of the activity difference between the chemicals in each pair for one receptor. The color gradient represents the similarity between chemicals in each pair (where the darker shade represents a higher structural similarity). (b) Chemical pairs forming triple-target cliffs, where the arrow denotes the direction of SAR. (c–e) DAD maps corresponding to each pair of receptors considered in the TAD map. The axis represents the receptors, and the color gradient represents the similarity between chemicals in each pair (where the darker shade represents higher structural similarity). (f) Chemical pairs forming dual-target cliffs, where the arrow denotes the direction of SAR. (g) Chemical pairs forming single-target cliffs with respect to each of the receptors considered in the TAD map.

We employed the TAD map- and DAD map-based approach to identify single-, dual-, and triple-target cliffs among the generated 56 triple-target and 28 dual-target combinations in the agonist data set (Methods section). The DAD map approach aids in the identification of chemical pairs forming activity cliffs against two targets (dual-target activity cliffs), whereas the TAD map approach, which is a combination of three DAD maps, additionally aids in the identification of chemical pairs forming activity cliffs against three targets (triple-target cliffs).^26^ For example, chemicals targeting AR, ERα, and ERβ showed the highest fraction of triple-target activity cliffs (13 triple-target cliffs among 200 chemical pairs; Figure 1a and Table S6). Notably, all 13 triple-target cliffs identified in the AR-ERα-ERβ TAD map show a common trend of inverse SAR with respect to AR-ERα and AR-ERβ targets and similar SAR with ERα-ERβ targets. These 13 triple-target cliffs are formed by 14 chemicals, and ClassyFire^27^ categorized all of these chemicals under the superclass “lipids and lipid-like molecules”. Figure 1b shows the triple-target cliffs formed by chemical pairs [CAS: 4245-41-4, CAS:434-22-0] and [CAS:68-22-4, CAS:965-90-2], where the direction of SAR with respect to the AR receptor is inverse to that of ERα and ERβ receptors.

We further split the AR-ERα-ERβ TAD map into three DAD maps, namely, AR-ERα (Figure 1c), AR-ERβ (Figure 1d), and ERα-ERβ (Figure 1e), to identify the dual-target and single-target cliffs. Among the three DAD maps, the AR-ERα DAD map showed the highest fraction of dual-target and single-target cliffs (91 dual-target cliffs and 174 single-target cliffs among 859 chemical pairs; Table S4). Among the 91 dual-target cliffs identified in the AR-ERα DAD map, 10 have similar SAR and the remaining have inverse SAR (activity switches). Notably, these 91 dual-target cliffs are formed by 51 chemicals, among which 46 chemicals are classified under the superclass “lipids and lipid-like molecules”. Figure 1f shows an example of a similar SAR dual-target cliff formed by the chemical pair [CAS:434-03-7, CAS:5630-53-5] with respect to AR and ERα receptors and an inverse SAR dual-target cliff (activity switch) formed by the chemical pair [CAS:297-76-7, CAS:965-90-2] with respect to AR and ERβ receptors. Figure 1g shows examples of single-target cliffs with respect to AR [CAS:10161-33-8, CAS:850-52-2], ERα [CAS:68047-06-3, CAS:68392-35-8], and ERβ [CAS:4245-41-4, CAS:57-91-0] receptors.

### Identification of Single-, Dual- and Triple-Target Activity Cliffs among Chemicals in the Antagonist Data Set

**Figure 2:**
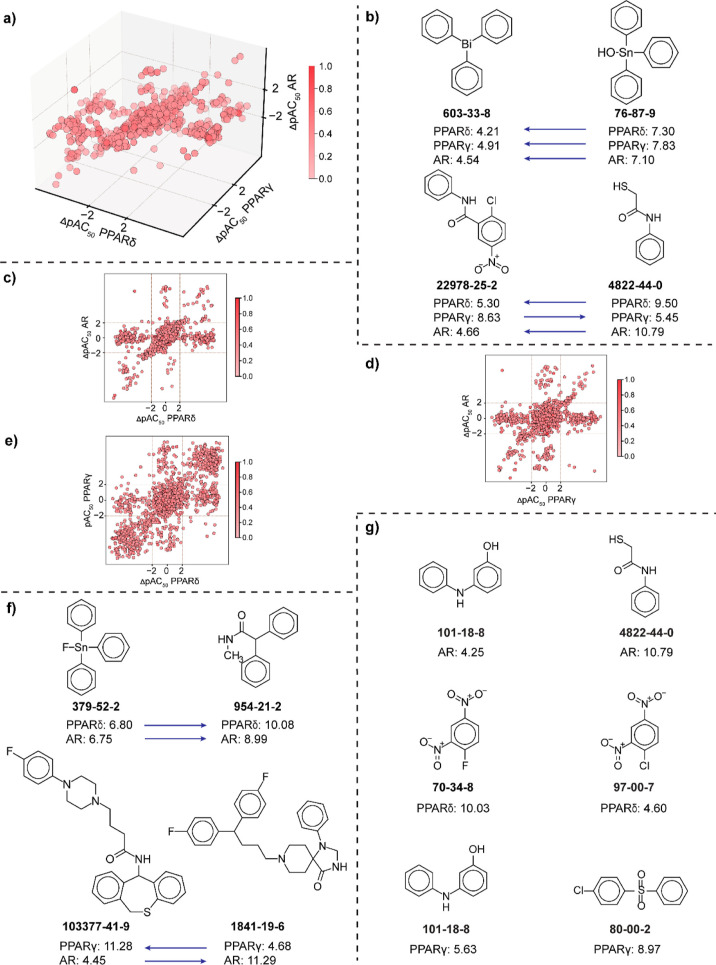
Analysis of the structure–activity landscape of chemicals in the antagonist data set. (a) TAD map for common and structurally similar chemicals targeting AR, PPARδ, and PPARγ receptors. Each axis of the TAD map represents the values of activity difference between the chemicals in each pair for one receptor. The color gradient represents the similarity between chemicals in each pair (where the darker shade represents higher structural similarity). (b) Chemical pairs forming triple-target cliffs, where the arrow denotes the direction of SAR. (c–e) DAD maps corresponding to each pair of receptors considered in the TAD map. The axis represents the receptors, and the color gradient represents the similarity between chemicals in each pair (where the darker shade represents a higher structural similarity). (f) Chemical pairs forming dual-target cliffs, where the arrow denotes the direction of SAR. (g) Chemical pairs forming single-target cliffs with respect to each of the receptors considered in the TAD map.

Similar to the agonist data set, we employed TAD and DAD map-based approach to identify the single-, dual-, and triple-target cliffs among the structurally similar chemicals from the 56 triple-target and 28 dual-target combinations in antagonist data set (Methods). Chemicals targeting AR, PPARδ, and PPARγ showed the highest fraction of triple-target activity cliffs (66 triple-target cliffs among 1060 chemical pairs; Figure 2a and Table S7). Notably, 62 of 66 triple-target cliffs identified in AR-PPARδ-PPARγ TAD map show a common trend of similar SAR with respect to AR-PPARδ, AR-PPARγ, and PPARδ-PPARγ targets. The 66 triple-target cliffs are formed by 60 chemicals, among which 43 are classified under the superclass “benzenoids”. Figure 2b shows triple-target cliffs formed by chemical pairs [CAS:603-33-8, CAS:76-87-9] with similar SAR with respect to all the 3 receptors (AR, PPARδ, and PPARγ) and [CAS:22978-25-2, CAS:4822-44-0], where the direction of SAR with respect to the PPARγ receptor is inverse to that of AR and PPARδ receptors.

Further, upon splitting the AR-PPARδ-PPARγ TAD map into three corresponding DAD maps (Figure 2c–e), we observed that the PPARδ-PPARγ DAD map showed the highest fraction of dual-target cliffs (621 out of 2226 pairs), while AR-PPARδ showed the highest fraction of single-target cliffs (353 out of 1521 pairs) (Table S5). Among the 621 dual-target cliffs from the PPARδ-PPARγ DAD map (Figure 2e), 608 show similar SAR and 13 show inverse SAR (activity switches). These 621 dual-target cliffs are formed by 342 chemicals, among which 198 chemicals are classified under the superclass “benzenoids”. The high percentage of similar SAR among PPARδ-PPARγ dual-target cliffs can be attributed to the high correlation between activity values of the chemicals targeting these two receptors. Figure 2f shows an example of a similar SAR dual-target cliff formed by the chemical pair [CAS:379-52-2, CAS:954-21-2] with respect to AR and PPARδ receptors and an inverse SAR dual-target cliff (activity switch) formed by the chemical pair [CAS:103377-41-9, CAS:1841-19-6] with respect to AR and PPARγ receptors. Figure 2g shows examples of single-target cliffs with respect to each of AR [CAS:101-18-8, CAS:4822-44-0], PPARδ [CAS:70-34-8, CAS:97-00-7], and PPARγ [CAS:101-18-8, CAS:80-00-2] receptors.

### MMP-Based Annotation of the Identified Activity Cliffs

**Figure 3:**
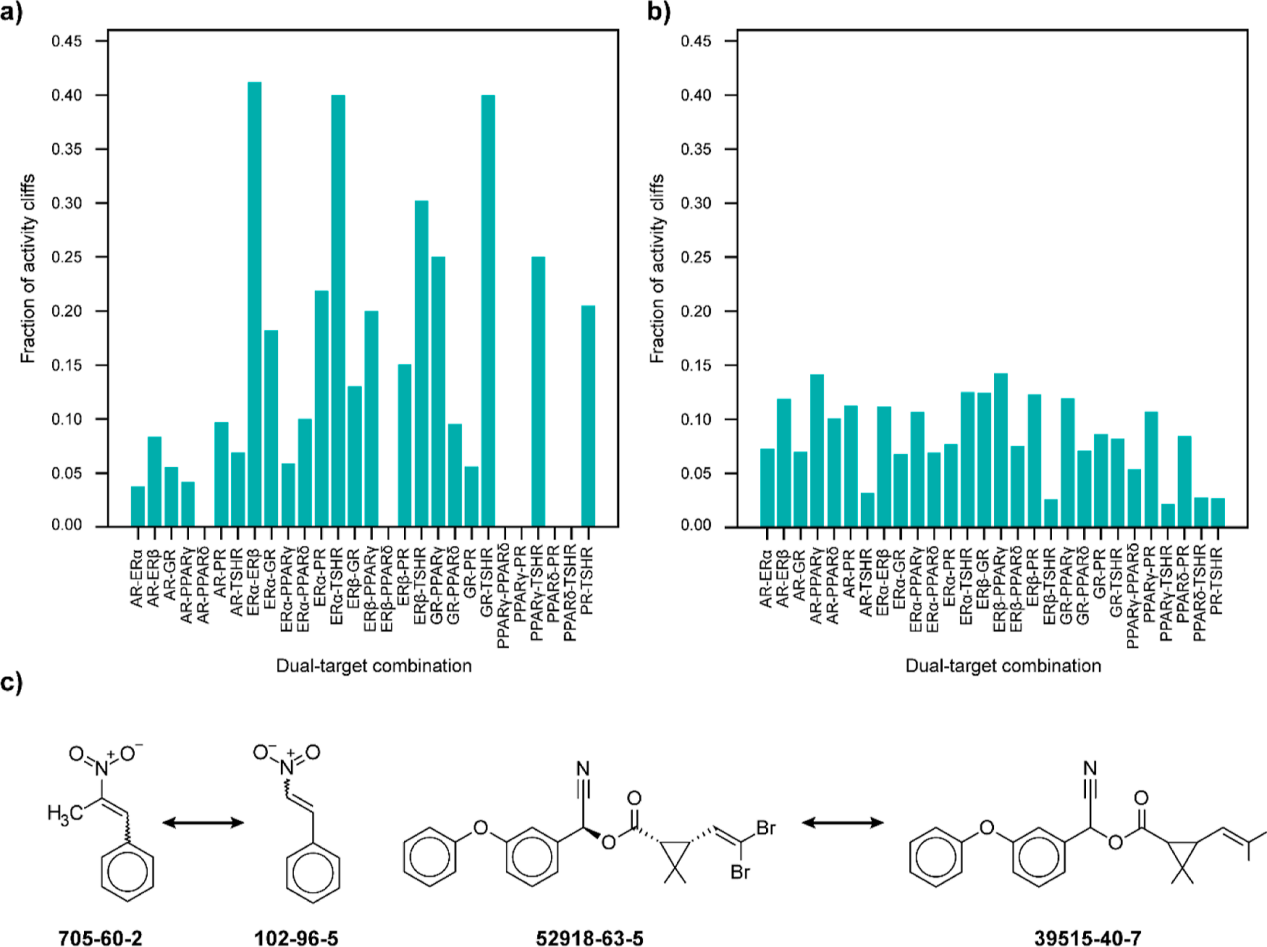
Exploration of MMPs among the activity cliffs identified via the DAD maps. (a) Distribution of MMPs among activity cliffs identified in the agonist data set via DAD maps. (b) Distribution of MMPs among activity cliffs identified in the antagonist data set via DAD maps. (c) Chemical pairs forming MMPs in agonist and antagonist data sets.

**Figure 4:**
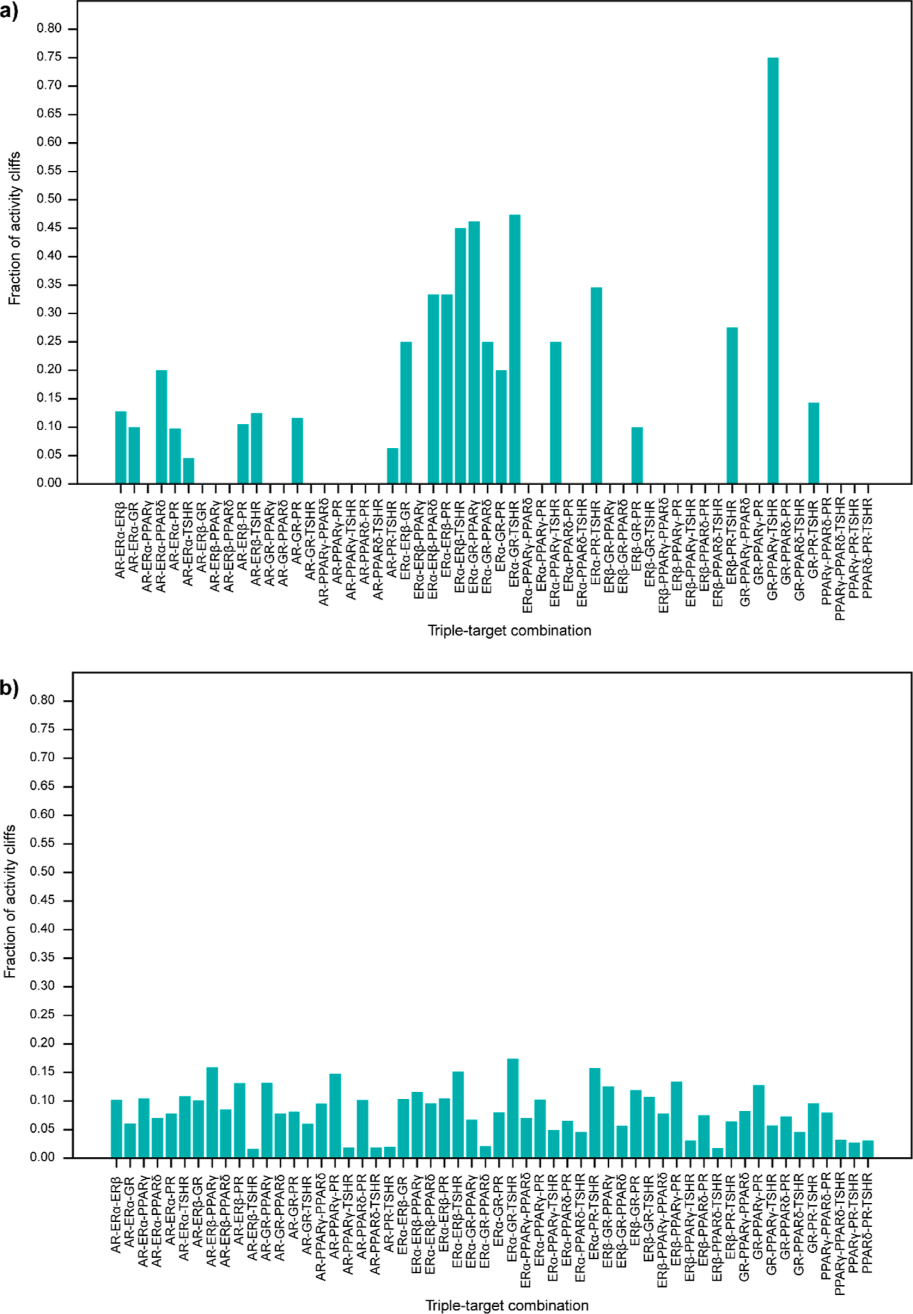
Exploration of MMPs among the activity cliffs identified via the TAD maps. (a) Distribution of MMPs among activity cliffs identified in the agonist data set via TAD maps. (b) Distribution of MMPs among activity cliffs identified in the antagonist data set via TAD maps.

We leveraged the MMP-based approach to annotate the activity cliffs identified in both the agonist and antagonist data sets via DAD and TAD maps (Methods section and Table S8). Among the different receptor combinations in the agonist data set, the dual-target combination of ERα-ERβ receptors (Figure 3a) shows the highest fraction of MMPs, while the triple-target combination of GR-PPARγ-TSHR receptors (Figure 4a) shows the highest fraction of MMPs. Similarly, among the different receptor combinations in the antagonist data set, the dual-target combination of ERβ-PPARγ receptors (Figure 3b) shows the highest fraction of MMPs, while the triple-target combination of ERα-GR-TSHR receptors (Figure 4b) shows the highest fraction of MMPs. Figure 3c displays the examples of MMPs formed by the dual-target combination of ERα-PPARγ in the agonist data set (CAS:705-60-2, CAS:102-96-5) and PPARδ-PR in the antagonist data set (CAS:52918-63-5, CAS:39515-40-7).

### Structural Classification of the Identified Activity Cliffs

**Figure 5:**
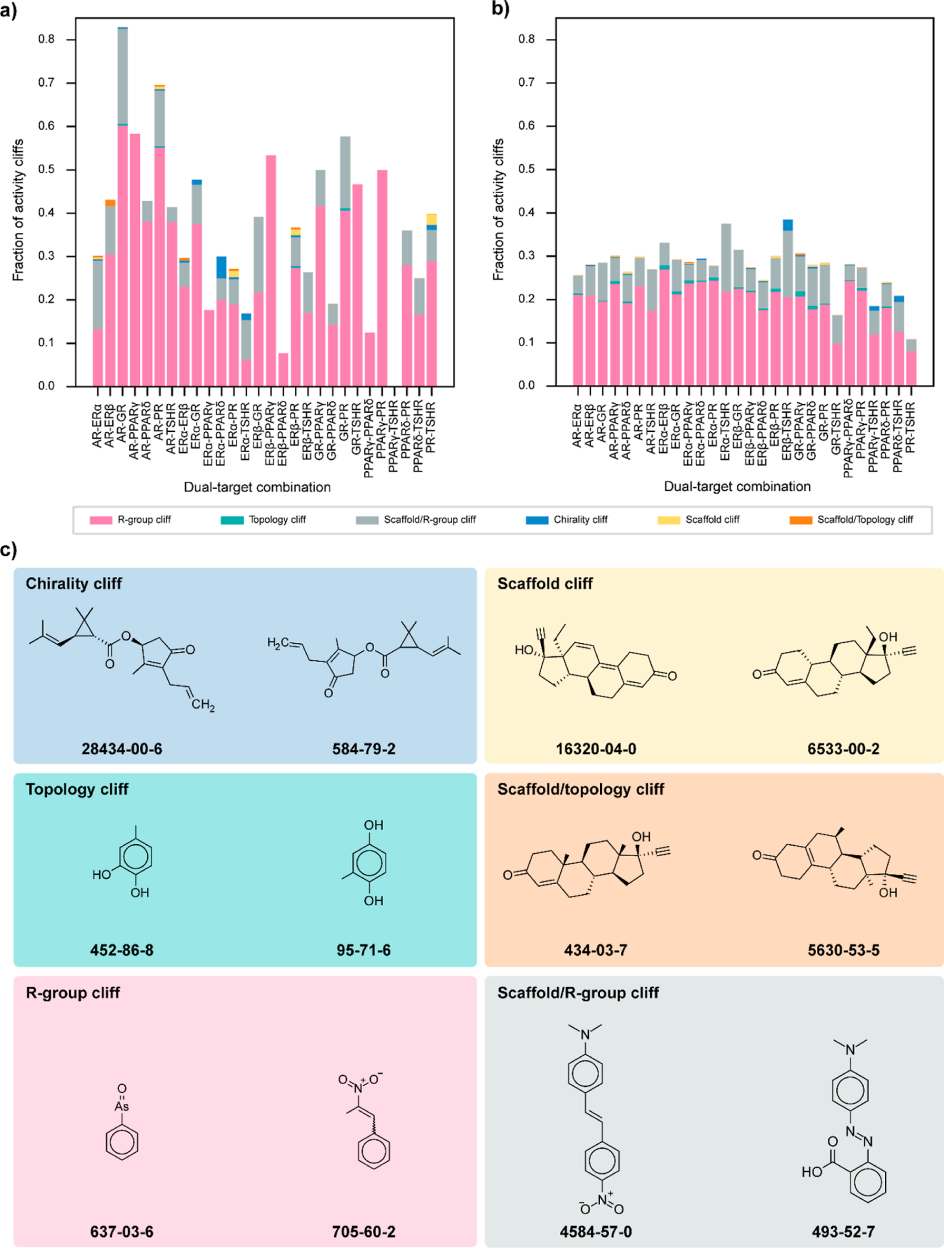
Classification of activity cliffs identified via DAD maps. (a) Distribution of different structural classifications among activity cliffs identified in the agonist data set via DAD maps. (b) Distribution of different structural classifications among activity cliffs identified in the antagonist data set via DAD maps. (c) Examples of activity cliff pairs that form different structural classes.

**Figure 6:**
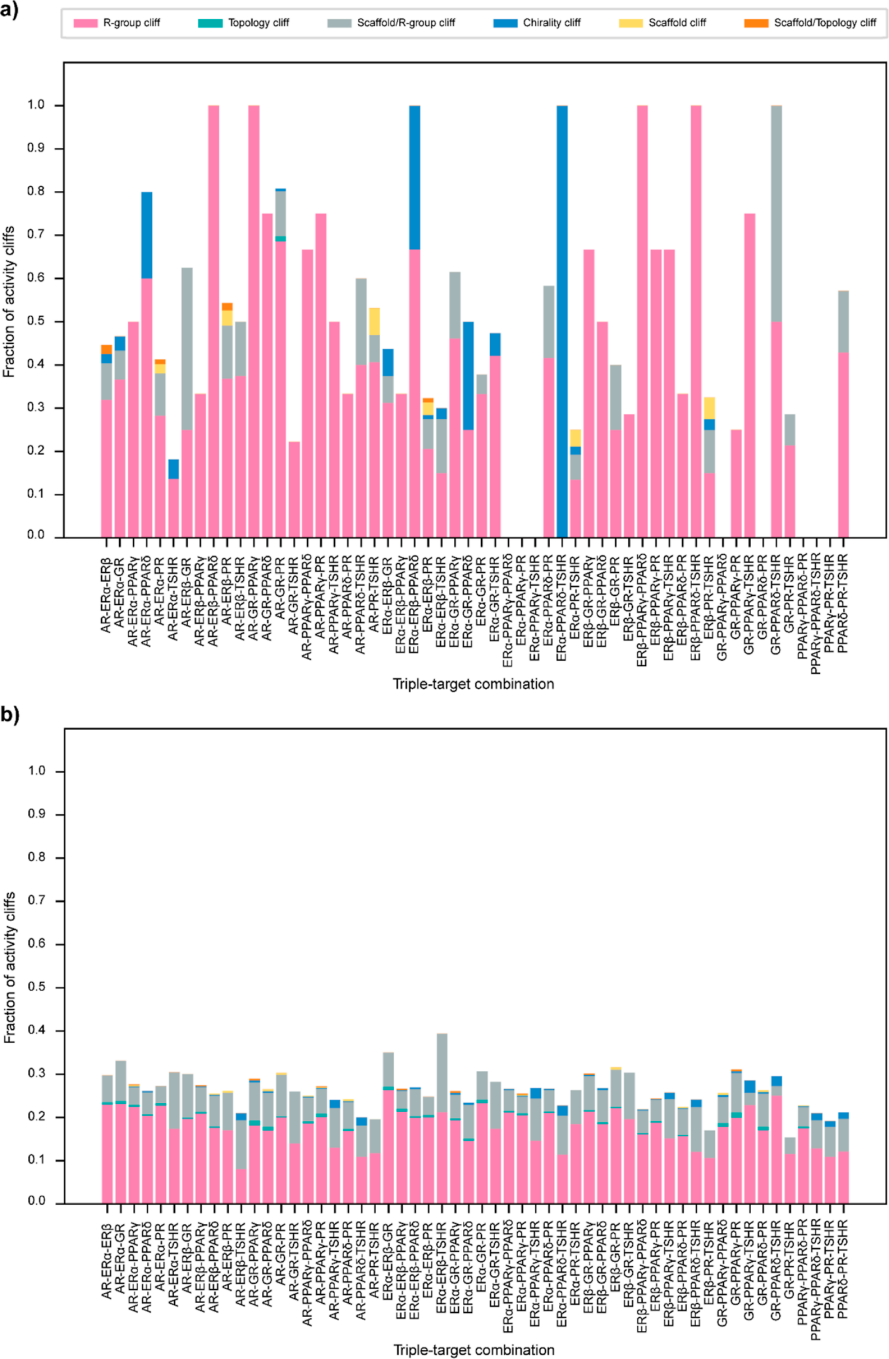
Classification of activity cliffs identified via the TAD maps. (a) Distribution of different structural classifications among activity cliffs identified in the agonist data set via TAD maps. (b) Distribution of different structural classifications among activity cliffs identified in the antagonist data set via TAD maps.

By leveraging the structural features of the chemicals, we independently classified the activity cliffs identified in agonist and antagonist data sets via DAD and TAD maps (Methods section and Table S9). We observed that in most of the dual-target or triple-target combinations, the fraction of the R-group cliffs is the highest. Among the different receptor combinations in the agonist data set, the dual-target combination of AR-PR and ERβ-PR showed all six structural classifications (Figure 5a), while the triple-target combination of ERα-ERβ-PR showed a maximum of 5 structural classifications (Figure 6a). Similarly, among the different combinations in the antagonist data set, the dual-target combinations of AR-PPARγ, ERα-PPARγ, and PPARγ-PR (Figure 5b) and the triple-target combinations of AR-ERα-PPARγ, AR-PPARγ-PR, and ERα-PPARγ-PR (Figure 6b) showed all 6 structural classifications. Figure 5c shows examples of chemical pairs forming 6 structural classifications, namely, chirality cliff (formed by CAS:28434-00-6 and CAS:584-79-2 across 13 DAD maps in the antagonist data set), topology cliff (formed by CAS:452-86-8 and CAS:95-71-6 across 15 DAD maps in the antagonist data set), R-group cliff (formed by CAS:637-03-6 and CAS:705-60-2 across 18 DAD maps in the antagonist data set), scaffold cliff (formed by CAS:16320-04-0 and CAS:6533-00-2 across 4 DAD maps in the agonist data set and 3 DAD maps in the antagonist data set), scaffold/topology cliff (formed by CAS:434-03-7 and CAS:5630-53-5 across 5 DAD maps in the agonist data set), and scaffold/R-group cliff (formed by CAS:4584-57-0 and CAS:493-52-7 across 15 DAD maps in the antagonist data set).

### Identification of Strong and Weak MOA-Cliffs

**Figure 7:**
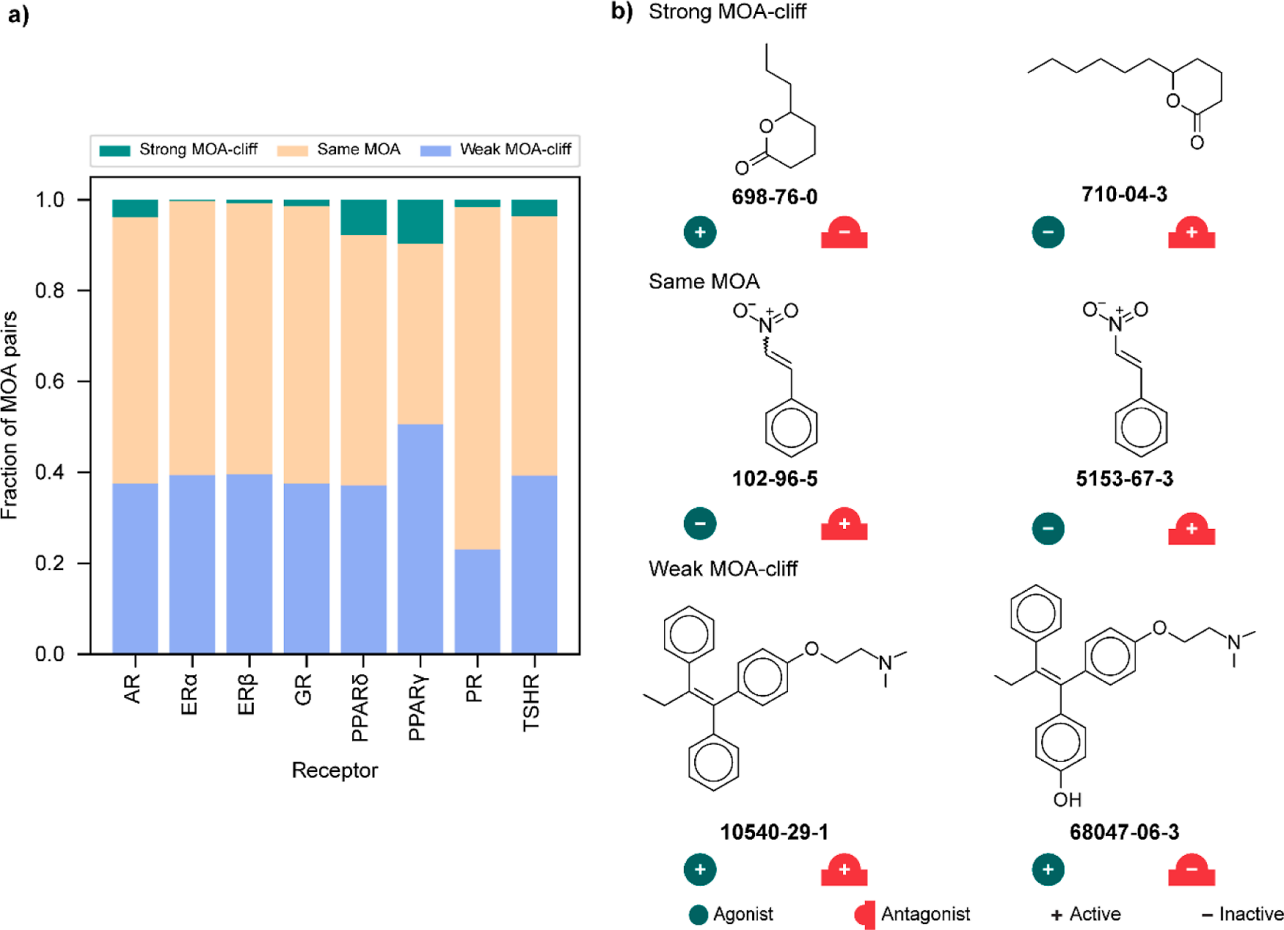
Identification of various MOA-cliffs. (a) Distribution of strong MOA-cliffs, weak MOA-cliffs, and the same MOA across chemicals targeting the eight human endocrine receptors. (b) Chemical pairs forming the strong MOA-cliff, same MOA, and weak MOA-cliff.

In addition to analyzing the structure–activity landscape, we also analyzed the heterogeneity in the structure–mechanism relationship of chemicals with respect to each of the eight human endocrine receptors. We shortlisted the common chemicals between agonist and antagonist data sets for each receptor and, thereafter, identified strong and weak MOA-cliffs using their MOA annotations (Methods section and Table S10). Figure 7a shows the distribution of the strong MOA-cliffs, weak MOA-cliffs, and the same MOA across each of the eight receptors. Among the eight receptors, PPARγ has the highest fraction of strong MOA-cliffs (8 of 83 MOA pairs) and weak MOA-cliffs (42 of 83 MOA pairs), while PR has the highest number of strong MOA-cliffs (54 MOA pairs) and weak MOA-cliffs (807 MOA pairs). Figure 7b displays examples of the strong MOA-cliff (formed by CAS:698-76-0 and CAS:710-04-3), the weak MOA-cliff (formed by CAS:10540-29-1 and CAS:68047-06-3), and the same MOA (formed by CAS:102-96-5 and CAS:5153-67-3) with respect to the PPARγ receptor.

## Conclusions

In this study, we systematically analyzed the structure–activity landscape of environmental chemicals targeting multiple human endocrine receptors. The activity values of chemicals targeting GR and PPARγ receptors in the agonist data set and PPARδ and PPARγ receptors in the antagonist data set showed the highest correlation, suggesting a similarity between their structure–activity landscapes. Further, we observed that AR-ERα-ERβ and AR-PPARδ-PPARγ TAD maps had the highest fraction of triple-target cliffs in the agonist and antagonist data sets, respectively. Importantly, we also structurally categorized the activity cliff pairs (single-, dual-, and triple-target cliff pairs) and observed that the R-group cliff category had the highest fraction among the activity cliffs identified from various target combinations. Finally, we leveraged the MOA annotations and identified the strong and weak MOA-cliffs among chemicals targeting each of the eight receptors. The present study is an example of the structure multiple activity relationships^28^ (SMARt) analysis performed on an environmental chemical space. To the best of our knowledge, this is the first study that explores and analyzes the structure–activity landscape of environmental chemicals targeting multiple human endocrine receptors.

The ToxCast chemical library is the largest chemical resource that has screened various environmental chemicals across different cell lines and quantitatively cataloged the corresponding biological interactions. Among the curated list of 3829 chemicals from ToxCast analyzed in this study, 312 chemicals have been cataloged as EDCs in DEDuCT^5^ and 474 chemicals are documented as high production volume (HPV) chemicals by the OECD HPV^29^ and US HPV.^30^ We also observed a low overlap of chemicals binding to all eight human endocrine receptors in each of the agonist (20 chemicals) and antagonist (100 chemicals) data sets. This suggests that the extent of promiscuity in the binding of environmental chemicals (assessed by the ToxCast project) to these eight endocrine receptors is low.

However, the present study does not address the molecular mechanisms underlying the formation of various multitarget activity cliffs due to the unavailability of the experimentally determined cocrystallized protein structures for the eight endocrine receptors with the chemicals forming activity cliffs. Further, we are restricted to only eight human endocrine receptors due to the lack of high-confidence data sets. Nonetheless, this study highlights the presence of multitarget activity cliffs among environmental chemicals targeting different human receptors. Overall, we expect that the findings from this study will aid in the development of well-informed machine learning-based toxicity prediction models^31^ with a broader applicability domain and contribute toward human chemical exposome research.

## Methods

### Curated Data Set of Chemicals Targeting Multiple Endocrine Receptors

In this study, our main objective is to analyze the activity landscape of chemicals that can target multiple endocrine receptors in humans. To this end, we leveraged the chemical data set from the high-throughput Tox21 assays (assay source identifier 7) with level 5 and 6 preprocessing within ToxCast version 3.5.^32^ Tox21 captures 146 assay end points spread across various cell lines and targets. To ensure a high-confidence chemical data set specific to human endocrine receptors, we filtered the Tox21 data to obtain assay end points that (i) are primary readouts; (ii) are performed on human cell lines; (iii) have corresponding agonist or antagonist end point annotations; (iv) are not follow-up assays; and (v) independently target a single human endocrine receptor. Based on these criteria, we identified assays corresponding to 16 end points across eight human endocrine receptors (Table S1).

Thereafter, we used an in-house R script to obtain the chemical information from ToxCast for these 16 assay end points. In particular, we filtered chemicals that were annotated as representative chemicals (gsid_rep = 1) and had a reported activity value (modl_ga is present). Note that we have considered only those chemicals for which the activity value is reported in ToxCast. Next, we accessed the two-dimensional (2D) structures of chemicals from ToxCast version 3.5 or PubChem.^33^ Further, we used MayaChemTools^34^ to remove the salts, invalid molecules, mixtures, and duplicated chemicals. Moreover, based on our previous studies,^16,17^ we computed the Bemis-Murcko scaffolds^35^ of chemicals and removed the linear molecules. Finally, we curated and compiled eight human endocrine receptor specific agonist data sets (Table S2) and antagonist data sets (Table S3) containing the CAS or PubChem identifiers, activity values, and end point annotations (active or inactive), which were further considered for various analyses.

### Computation of Chemical Similarity and Activity Difference

We computed the structural similarity between any pair of chemicals based on the Tanimoto coefficient of their corresponding ECFP4^36,37^ (extended connectivity fingerprints with diameter 4) chemical fingerprints. Note that we used the ECFP4 fingerprint since it is one of the best performing fingerprints in capturing the structural similarity^38^ and has been extensively used in various literature studies to analyze the structure–activity landscape of diverse chemical data sets.^16,17,39,40^ The activity difference between any pair of chemicals is given by the difference in their pAC_50_ values, where pAC_50_ is the negative logarithm of the AC_50_ value in molar concentration. The obtained chemical data sets from ToxCast consist of chemical activities mentioned in terms of modl_ga values, which is the logarithm of AC_50_ value in micromolar concentration. We used the following formulas to obtain the corresponding pAC_50_ value of chemicals
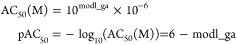


Finally, we computed the activity difference between two chemicals (*i*, *j*) against a particular target *T* using the following formula
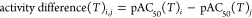


### Identification of Activity Cliffs Based on DAD Maps and TAD Maps

**Figure 8:**
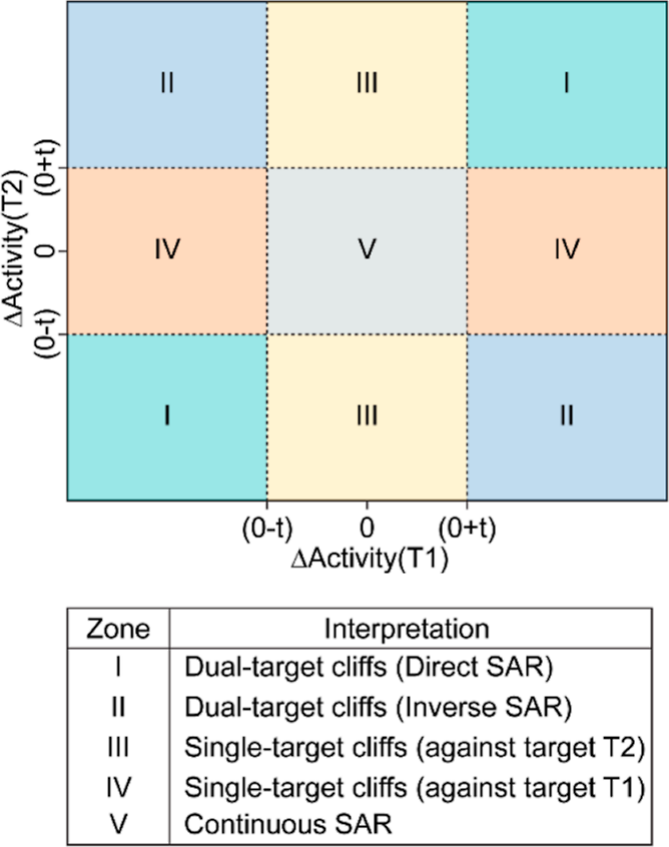
Prototype of the DAD map against targets T1 and T2, where ‘*t*’ is the activity difference threshold. The DAD map can be divided into 5 zones (*I*–*V*), and the interpretation of the 5 zones is tabulated below the DAD map.

We independently analyzed the SAR of chemicals in agonist and antagonist data sets using the DAD maps and TAD maps.^25,26^ A DAD map is a 2D representation where the axes denote the activity difference between chemicals against two different targets and each point on the plot denotes a chemical pair (Figure 8). We obtained all possible combinations of targets from each data set and considered the common chemicals in these combinations (Tables S4 and S5). Further, in each of these combinations, we obtained structurally similar chemical pairs as those having a similarity value greater than or equal to three standard deviations from the median of the similarity distribution (Tables S4–S7). For each of these chemical pairs, we computed their activity difference by preserving their sign and plotted them on a DAD map. To identify significant differences in activity values, we set an activity difference threshold of −2 and 2 on each axis and divided the DAD map into 5 zones, namely, zone I to zone V (Figure 8). The chemical pairs in zone I show a similar trend of differences in activity ([ΔpAC_50_(T1) and ΔpAC_50_(T2)] > 2 or < −2) against both targets, denoting a similar SAR. The chemical pairs in zone II show an inverse trend of differences in activity (ΔpAC_50_(T1) > 2 and ΔpAC_50_(T2) < −2, or vice versa) against the two targets, denoting an inverse SAR. Note that the chemical pairs falling in zone II can be considered as activity switches.^41^ Additionally, chemical pairs in zone I and zone II are referred to as dual-target cliffs.^26^ The chemical pairs in zone III and zone IV show a significant difference in activity against only one of the two targets and are referred to as single-target cliffs.^26^ The chemical pairs in zone V show no significant difference in activity against either of the targets and hence do not form activity cliffs.

**Figure 9:**
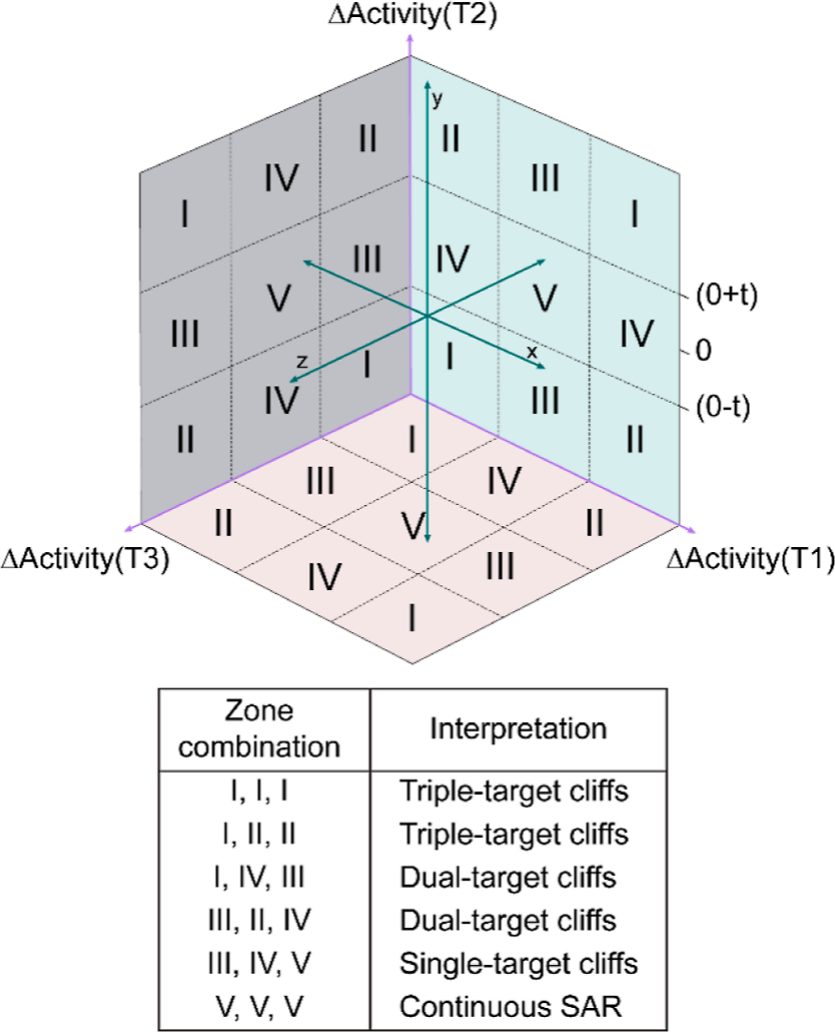
Prototype of the TAD map against targets T1, T2, and T3, where ‘*t*’ is the activity difference threshold. The interpretation of the various zone combinations of the TAD map is tabulated below the TAD map.

A TAD map is a 3D representation where the axes denote the activity difference between chemicals against three different targets and each point on the plot denotes a chemical pair (Figure 9). Similar to the DAD map approach, we obtained structurally similar chemical pairs for all possible combinations of three targets and computed their corresponding differences in activity values. To identify significant differences in activity values, we set an activity difference threshold of +2 and −2 along each axis and identified single-target, dual-target and triple-target cliffs (Figure 9).

### Annotation of Activity Cliffs Based on MMPs

MMPs are chemical pairs that structurally differ at a single site. It is a substructure-based approach that is descriptor-independent, metric-free, and chemically intuitive.^42^ Therefore, we employed the MMP-based approach^43^ to annotate the activity cliffs identified from DAD and TAD maps. Based on our previous work,^17^ we employed the mmpdb platform^44^ to generate MMPs for each of the 16 data sets. First, we performed the fragmentation of the chemicals using the mmpdb fragmentation module with “none” value for both the maximum number of non-hydrogen atoms and the maximum number of rotatable bonds arguments. Then, we generated an exhaustive list of MMPs by using the mmpdb index module with “none” value for the maximum number of non-hydrogen atoms in the variable fragment argument. Further, we obtained the size-restricted MMPs using the following criteria:^17^

(i) The difference in the number of heavy atoms of the exchanged fragments in the transformation should be ≤8.
(ii) The constant part of an MMP should be at least twice the size of each fragment in the transformation.
(iii) The number of heavy atoms (non-hydrogen atoms) of each fragment in the transformation should be ≤13.
(iv) If a chemical pair has multiple MMPs, the transformation that has the least heavy atom difference in the exchanged fragments is considered.

### Structural Classification of Activity Cliffs

We provided structural classification of activity cliffs identified from DAD and TAD maps by considering information on molecular scaffolds, R-groups, R-group topology, and chirality of chemical structures.^15^ Based on our previous work,^17^ we developed a python workflow that employs RDKit^45^ to classify the activity cliffs into the following seven classes:

(i) Chirality cliff: activity cliff pairs whose scaffolds, R-groups, and R-group topologies are the same.
(ii) Topology cliff: activity cliff pairs whose R-group topologies are different, while their scaffolds and R-groups are the same.
(iii) R-group cliff: activity cliff pairs whose R-groups are different, while their scaffolds are the same.
(iv) Scaffold cliff: activity cliff pairs whose scaffolds are different, while their cyclic skeletons, R-groups, and R-group topologies are the same.
(v) Scaffold/topology cliff: activity cliff pairs whose scaffolds and R-group topologies are different, while their cyclic skeletons and R-groups are the same.
(vi) Scaffold/R-group cliff: activity cliff pairs whose scaffolds and R-groups are different, while their cyclic skeletons are the same.
(vii) Unclassified: activity cliff pairs whose both scaffolds and cyclic skeletons are different.

### Analysis of the Structure–Mechanism Relationships of Endocrine Receptor Binding Chemicals

Apart from the heterogeneity in their structure–activity landscape, chemicals can show heterogeneity in their structure–mechanism relationships leading to MOA-cliffs.^17,42^ Based on our previous work,^17^ we first identified the common chemicals between the agonist and antagonist data sets for each of the eight human receptors. Then, for each receptor, we filtered out the chemicals that were inactive in both the agonist and antagonist assay end points (hit_c value is 0) and shortlisted structurally similar chemical pairs whose Tanimoto coefficient was greater than or equal to 3 standard deviations from the median of the similarity distribution. Finally, based on the MOA annotations, we classified the chemical pairs (MOA pairs) into three categories:

(i) Strong MOA-cliff: chemical pairs for which the MOA annotations are the opposite in both agonist and antagonist assay end points.
(ii) Same MOA: chemical pairs for which MOA annotations are the same in both agonist and antagonist assay end points.
(iii) Weak MOA-cliff: chemical pairs which could not be categorized as either Strong MOA-cliff or Same MOA.

## Supporting Information Available

Curated list of eight human endocrine receptors; curated list of chemicals in the agonist data set for the eight receptors from the ToxCast chemical library; curated list of chemicals in the antagonist data set for the eight receptors from the ToxCast chemical library; list of 28 DAD maps formed by dual-target combinations among the eight receptors in the agonist data set; list of 28 DAD maps formed by dual-target combinations among the eight receptors in the antagonist data set; list of 56 TAD maps formed by triple-target combinations among the eight receptors in the agonist data set; list of 56 TAD maps formed by triple-target combinations among the eight receptors in the antagonist data set; list of MMPs identified among the chemicals in agonist or antagonist data sets; list of activity cliffs and their structural classification identified among the chemicals in agonist or antagonist data sets; and MOA-based classification of chemicals targeting each of the eight receptors (XLSX)

## References

[ref1] Wild C. P. Complementing the Genome with an “Exposome”: The Outstanding Challenge of Environmental Exposure Measurement in Molecular Epidemiology. Cancer Epidemiol. Biomarkers Prev. 2005, 14 (8), 1847–1850. 10.1158/1055-9965.EPI-05-0456. 16103423

[ref2] Diamanti-Kandarakis E.; Bourguignon J.-P.; Giudice L. C.; Hauser R.; Prins G. S.; Soto A. M.; Zoeller R. T.; Gore A. C. Endocrine-Disrupting Chemicals: An Endocrine Society Scientific Statement. Endocr. Rev. 2009, 30 (4), 293–342. 10.1210/er.2009-0002. 19502515 PMC2726844

[ref3] Gore A. C.; Chappell V. A.; Fenton S. E.; Flaws J. A.; Nadal A.; Prins G. S.; Toppari J.; Zoeller R. T. EDC-2: The Endocrine Society’s Second Scientific Statement on Endocrine-Disrupting Chemicals. Endocr. Rev. 2015, 36 (6), E1–E150. 10.1210/er.2015-1010. 26544531 PMC4702494

[ref4] Karthikeyan B. S.; Ravichandran J.; Mohanraj K.; Vivek-Ananth R. P.; Samal A. A Curated Knowledgebase on Endocrine Disrupting Chemicals and Their Biological Systems-Level Perturbations. Sci. Total Environ. 2019, 692, 281–296. 10.1016/j.scitotenv.2019.07.225. 31349169

[ref5] Karthikeyan B. S.; Ravichandran J.; Aparna S. R.; Samal A. DEDuCT 2.0: An Updated Knowledgebase and an Exploration of the Current Regulations and Guidelines from the Perspective of Endocrine Disrupting Chemicals. Chemosphere 2021, 267, 128898 10.1016/j.chemosphere.2020.128898. 33190914

[ref6] Casals-Casas C.; Desvergne B. Endocrine Disruptors: From Endocrine to Metabolic Disruption. Annu. Rev. Physiol. 2011, 73 (1), 135–162. 10.1146/annurev-physiol-012110-142200. 21054169

[ref7] Nobeli I.; Favia A. D.; Thornton J. M. Protein Promiscuity and Its Implications for Biotechnology. Nat. Biotechnol. 2009, 27 (2), 157–167. 10.1038/nbt1519. 19204698

[ref8] Kabir A.; Muth A. Polypharmacology: The Science of Multi-Targeting Molecules. Pharmacol. Res. 2022, 176, 106055 10.1016/j.phrs.2021.106055. 34990865

[ref9] Lo Piparo E.; Siragusa L.; Raymond F.; Passeri G. I.; Cruciani G.; Schilter B. Bisphenol A Binding Promiscuity: A Virtual Journey through the Universe of Proteins. ALTEX 2020, 37 (1), 85–94. 10.14573/altex.1906141. 31707420

[ref10] Dix D. J.; Houck K. A.; Martin M. T.; Richard A. M.; Setzer R. W.; Kavlock R. J. The ToxCast Program for Prioritizing Toxicity Testing of Environmental Chemicals. Toxicol. Sci. 2007, 95 (1), 5–12. 10.1093/toxsci/kfl103. 16963515

[ref11] Richard A. M.; Judson R. S.; Houck K. A.; Grulke C. M.; Volarath P.; Thillainadarajah I.; Yang C.; Rathman J.; Martin M. T.; Wambaugh J. F.; Knudsen T. B.; Kancherla J.; Mansouri K.; Patlewicz G.; Williams A. J.; Little S. B.; Crofton K. M.; Thomas R. S. ToxCast Chemical Landscape: Paving the Road to 21st Century Toxicology. Chem. Res. Toxicol. 2016, 29 (8), 1225–1251. 10.1021/acs.chemrestox.6b00135. 27367298

[ref12] Richard A. M.; Huang R.; Waidyanatha S.; Shinn P.; Collins B. J.; Thillainadarajah I.; Grulke C. M.; Williams A. J.; Lougee R. R.; Judson R. S.; Houck K. A.; Shobair M.; Yang C.; Rathman J. F.; Yasgar A.; Fitzpatrick S. C.; Simeonov A.; Thomas R. S.; Crofton K. M.; Paules R. S.; Bucher J. R.; Austin C. P.; Kavlock R. J.; Tice R. R. The Tox21 10K Compound Library: Collaborative Chemistry Advancing Toxicology. Chem. Res. Toxicol. 2021, 34 (2), 189–216. 10.1021/acs.chemrestox.0c00264. 33140634 PMC7887805

[ref13] Guha R.; Van Drie J. H. Structure-Activity Landscape Index: Identifying and Quantifying Activity Cliffs. J. Chem. Inf. Model. 2008, 48 (3), 646–658. 10.1021/ci7004093. 18303878

[ref14] Medina-Franco J. L.; Martínez-Mayorga K.; Bender A.; Marín R. M.; Giulianotti M. A.; Pinilla C.; Houghten R. A. Characterization of Activity Landscapes Using 2D and 3D Similarity Methods: Consensus Activity Cliffs. J. Chem. Inf. Model. 2009, 49 (2), 477–491. 10.1021/ci800379q. 19434846

[ref15] Hu Y.; Bajorath J. Extending the Activity Cliff Concept: Structural Categorization of Activity Cliffs and Systematic Identification of Different Types of Cliffs in the ChEMBL Database. J. Chem. Inf. Model. 2012, 52 (7), 1806–1811. 10.1021/ci300274c. 22758389

[ref16] Vivek-Ananth R. P.; Sahoo A. K.; Baskaran S. P.; Ravichandran J.; Samal A. Identification of Activity Cliffs in Structure-Activity Landscape of Androgen Receptor Binding Chemicals. Sci. Total Environ. 2023, 873, 162263 10.1016/j.scitotenv.2023.162263. 36801331

[ref17] Sahoo A. K.; Baskaran S. P.; Chivukula N.; Kumar K.; Samal A. Analysis of Structure-Activity and Structure-Mechanism Relationships among Thyroid Stimulating Hormone Receptor Binding Chemicals by Leveraging the ToxCast Library. RSC Adv. 2023, 13 (34), 23461–23471. 10.1039/D3RA04452A. 37546222 PMC10401517

[ref18] Wassermann A. M.; Peltason L.; Bajorath J. Computational Analysis of Multi-Target Structure-Activity Relationships to Derive Preference Orders for Chemical Modifications toward Target Selectivity. ChemMedChem 2010, 5 (6), 847–858. 10.1002/cmdc.201000064. 20414918

[ref19] Hu Y.; Bajorath J. Molecular Scaffolds with High Propensity to Form Multi-Target Activity Cliffs. J. Chem. Inf. Model. 2010, 50 (4), 500–510. 10.1021/ci100059q. 20361784

[ref20] Hu Y.; Bajorath J. Introduction of Target Cliffs as a Concept To Identify and Describe Complex Molecular Selectivity Patterns. J. Chem. Inf. Model. 2013, 53 (3), 545–552. 10.1021/ci300602m. 23379346

[ref21] Dimova D.; Bajorath J. Rationalizing Promiscuity Cliffs. ChemMedChem 2018, 13 (6), 490–494. 10.1002/cmdc.201700535. 29024534

[ref22] Peltason L.; Hu Y.; Bajorath J. From Structure-Activity to Structure-Selectivity Relationships: Quantitative Assessment, Selectivity Cliffs, and Key Compounds. ChemMedChem 2009, 4 (11), 1864–1873. 10.1002/cmdc.200900300. 19750525

[ref23] Dimova D.; Wawer M.; Wassermann A. M.; Bajorath J. Design of Multitarget Activity Landscapes That Capture Hierarchical Activity Cliff Distributions. J. Chem. Inf. Model. 2011, 51 (2), 258–266. 10.1021/ci100477m. 21275393

[ref24] Waddell J.; Medina-Franco J. L. Bioactivity Landscape Modeling: Chemoinformatic Characterization of Structure-Activity Relationships of Compounds Tested across Multiple Targets. Bioorg. Med. Chem. 2012, 20 (18), 5443–5452. 10.1016/j.bmc.2011.11.051. 22178187

[ref25] Pérez-Villanueva J.; Santos R.; Hernández-Campos A.; Giulianotti M. A.; Castillo R.; Medina-Franco J. L. Structure-Activity Relationships of Benzimidazole Derivatives as Antiparasitic Agents: Dual Activity-Difference (DAD) Maps. Med. Chem. Commun. 2011, 2 (1), 44–49. 10.1039/C0MD00159G.

[ref26] Medina-Franco J. L.; Yongye A. B.; Pérez-Villanueva J.; Houghten R. A.; Martínez-Mayorga K. Multitarget Structure-Activity Relationships Characterized by Activity-Difference Maps and Consensus Similarity Measure. J. Chem. Inf. Model. 2011, 51 (9), 2427–2439. 10.1021/ci200281v. 21842860

[ref27] Djoumbou Feunang Y.; Eisner R.; Knox C.; Chepelev L.; Hastings J.; Owen G.; Fahy E.; Steinbeck C.; Subramanian S.; Bolton E.; Greiner R.; Wishart D. S. ClassyFire: Automated Chemical Classification with a Comprehensive, Computable Taxonomy. J. Cheminf. 2016, 8 (1), 61 10.1186/s13321-016-0174-y. PMC5096306 27867422

[ref28] Saldívar-González F. I.; Naveja J. J.; Palomino-Hernández O.; Medina-Franco J. L. Getting SMARt in Drug Discovery: Chemoinformatics Approaches for Mining Structure-Multiple Activity Relationships. RSC Adv. 2017, 7 (2), 632–641. 10.1039/C6RA26230A.

[ref29] 2004 OECD List of High Production Volume Chemicals. 2004. https://www.oecd.org/chemicalsafety/risk-assessment/33883530.pdf (accessed 2023–08–01).

[ref30] EPA. High Production Volume List. 2017. https://comptox.epa.gov/dashboard/chemical-lists/EPAHPV (accessed 2023–08–01).

[ref31] Liu W.; Wang Z.; Chen J.; Tang W.; Wang H. Machine Learning Model for Screening Thyroid Stimulating Hormone Receptor Agonists Based on Updated Datasets and Improved Applicability Domain Metrics. Chem. Res. Toxicol. 2023, 36 (6), 947–958. 10.1021/acs.chemrestox.3c00074. 37209109

[ref32] EPA. U. S. ToxCast & Tox21 Summary Files from invitrodb_v3., 2023 Retrieved from https://www.epa.gov/chemical-research/toxicity-forecaster-toxcasttm-data on April 4, 2023. Data released August 2022.

[ref33] PubChem. https://pubchem.ncbi.nlm.nih.gov/ (accessed 2023–08–01), 2023.

[ref34] Sud M. MayaChemTools: An Open Source Package for Computational Drug Discovery. J. Chem. Inf. Model. 2016, 56 (12), 2292–2297. 10.1021/acs.jcim.6b00505. 28024397

[ref35] Bemis G. W.; Murcko M. A. The Properties of Known Drugs. 1. Molecular Frameworks. J. Med. Chem. 1996, 39 (15), 2887–2893. 10.1021/jm9602928. 8709122

[ref36] Morgan H. L. The Generation of a Unique Machine Description for Chemical Structures-A Technique Developed at Chemical Abstracts Service. J. Chem. Doc. 1965, 5 (2), 107–113. 10.1021/c160017a018.

[ref37] Rogers D.; Hahn M. Extended-Connectivity Fingerprints. J. Chem. Inf. Model. 2010, 50 (5), 742–754. 10.1021/ci100050t. 20426451

[ref38] O’Boyle N. M.; Sayle R. A. Comparing Structural Fingerprints Using a Literature-Based Similarity Benchmark. J. Cheminf. 2016, 8 (1), 36 10.1186/s13321-016-0148-0. PMC4932683 27382417

[ref39] Naveja J. J.; Norinder U.; Mucs D.; López-López E.; Medina-Franco J. L. Chemical Space, Diversity and Activity Landscape Analysis of Estrogen Receptor Binders. RSC Adv. 2018, 8 (67), 38229–38237. 10.1039/C8RA07604A. 35559115 PMC9089822

[ref40] Naveja J. J.; Medina-Franco J. L. Activity Landscape Sweeping: Insights into the Mechanism of Inhibition and Optimization of DNMT1 Inhibitors. RSC Adv. 2015, 5 (78), 63882–63895. 10.1039/C5RA12339A.

[ref41] Medina-Franco J. L.; Edwards B. S.; Pinilla C.; Appel J. R.; Giulianotti M. A.; Santos R. G.; Yongye A. B.; Sklar L. A.; Houghten R. A. Rapid Scanning Structure-Activity Relationships in Combinatorial Data Sets: Identification of Activity Switches. J. Chem. Inf. Model. 2013, 53 (6), 1475–1485. 10.1021/ci400192y. 23705689 PMC3715655

[ref42] Hao M.; Bryant S. H.; Wang Y. Cheminformatics Analysis of the AR Agonist and Antagonist Datasets in PubChem. J. Cheminf. 2016, 8 (1), 37 10.1186/s13321-016-0150-6. PMC4938998 27398098

[ref43] Hu X.; Hu Y.; Vogt M.; Stumpfe D.; Bajorath J. MMP-Cliffs: Systematic Identification of Activity Cliffs on the Basis of Matched Molecular Pairs. J. Chem. Inf. Model. 2012, 52 (5), 1138–1145. 10.1021/ci3001138. 22489665

[ref44] Dalke A.; Hert J.; Kramer C. Mmpdb: An Open-Source Matched Molecular Pair Platform for Large Multiproperty Data Sets. J. Chem. Inf. Model. 2018, 58 (5), 902–910. 10.1021/acs.jcim.8b00173. 29770697

[ref45] RDKit: Open-Source Cheminformatics. 2023. https://www.rdkit.org/ (accessed 2023–08–01).

